# Shifting the Cancer Screening Paradigm: Developing a Multi-Biomarker Class Approach to Multi-Cancer Early Detection Testing

**DOI:** 10.3390/life14080925

**Published:** 2024-07-24

**Authors:** John B. Kisiel, Jon O. Ebbert, William R. Taylor, Catherine R. Marinac, Omair A. Choudhry, Seema P. Rego, Tomasz M. Beer, Michelle A. Beidelschies

**Affiliations:** 1 Mayo Clinic, Rochester, MN 55905, USA; kisiel.john@mayo.edu (J.B.K.); ebbert.jon@mayo.edu (J.O.E.); wtaylor@mayo.edu (W.R.T.); 2 Dana-Farber Cancer Institute, Boston, MA 02215, USA; catheriner_marinac@dfci.harvard.edu; 3 Exact Sciences Corporation, Madison, WI 53719, USA; ochoudhry@exactsciences.com (O.A.C.); srego@exactsciences.com (S.P.R.); tbeer@exactsciences.com (T.M.B.)

**Keywords:** liquid biopsy, biomarkers, tumor, early detection of cancer, DNA methylation, DNA mutational analysis, cell-free nucleic acids

## Abstract

Guideline-recommended screening programs exist for only a few cancer types. Although all these programs are understood to lead to reductions in cancer-related mortality, standard-of-care screening tests vary in accuracy, adherence and effectiveness. Recent advances in high-throughput technologies and machine learning have facilitated the development of blood-based multi-cancer cancer early detection (MCED) tests. MCED tests are positioned to be complementary to standard-of-care screening and they may broaden screening availability, especially for individuals who are not adherent with current screening programs and for individuals who may harbor cancers with no available screening options. In this article, we outline some key features that should be considered for study design and MCED test development, provide an example of the developmental pathway undertaken for an emerging multi-biomarker class MCED test and propose a clinical algorithm for an imaging-based diagnostic resolution strategy following MCED testing.

## 1. Introduction

Cancer is the second leading cause of death both in the United States (U.S.) and worldwide [1]. In the U.S., over 2 million new cancer cases and over 611,000 cancer deaths are projected to occur in 2024 [1]. Worldwide, there were over 19 million incident cases and almost 10 million cancer deaths in 2020, and the global cancer burden is expected to grow to 28.4 million cases by 2040 [2]. Compounding this epidemic are health disparities in cancer incidence, prevalence, morbidity and mortality due to factors such as age, race/ethnicity, gender, health literacy, genetics, income and geographic location [3].

To address the U.S. burden, the White House reignited the Cancer Moonshot^SM^ initiative in February 2022, which was initially launched in 2016 to accelerate the rate of progress against cancer by bringing together advocacy groups, patients, research institutions and healthcare communities [4]. This reignition established a new national goal to reduce mortality from cancer by at least 50 percent over the next 25 years [5]. Innovative technologies to detect, prevent and treat cancer are needed to achieve this goal. 

## 2. The Current State of Cancer Screening—A Single-Cancer Approach 

At present, United States Preventive Services Task Force (USPSTF)-endorsed screening programs in the U.S. exist for four cancer types—colorectal, breast, cervical and lung [6,7,8,9] (Table 1). These single-cancer screening programs are intended for the general population, with the exception of lung cancer screening which is intended for high-risk populations (i.e., adults aged 50 to 80 years with a 20 pack-year smoking history and currently smoke or have quit within the past 15 years). They also target various age groups and have distinct testing intervals. These screening programs leverage the imaging of tissues or a specific organ(s) of interest (i.e., mammography for breast cancer and low dose CT scan for lung cancer) combined with cytological testing and molecular photocopying (i.e., Papanicolaou test with human papillomavirus polymerase chain reaction testing) and both direct visualization (i.e., colonoscopy, flexible sigmoidoscopy, etc.) and non-invasive, stool-based modalities (i.e., Fecal Immunochemical Test (FIT), multi-target stool DNA (mt-sDNA), high-sensitivity guaiac fecal occult blood test (HS-gFOBT).

While current single-cancer screening programs have generally benefited the U.S. population, two-thirds of incident cancers and cancer deaths are the result of cancers without endorsed screening programs [1]. Moreover, current screening programs have several limitations. First, single-cancer screening programs focus on one organ of cancer origin at a time, thereby excluding many lower-prevalence cancers [11,12]. To illustrate this point, one can consider that screening for individual gastrointestinal cancers with prevalences of <1% (including esophageal, stomach, liver and pancreatic), would result in a number needed to screen ranging from 500 to 1000 persons to detect a single cancer case [11]. However, if all cancers were screened for in aggregate, using an overall cancer prevalence estimate of up to 3%, the number needed to screen would drop to around 33 when assuming 100% sensitivity [11]. Single-cancer screening for less common cancers would also require tests with extraordinarily high specificities to avoid an excess burden of false positives, as well as very large clinical trials to demonstrate clinical utility, which would likely be unachievable in most settings [13]. 

Current single-cancer screening programs have a significant likelihood of false positive results. The reported false positive rates for single-cancer tests are estimated at 11.1% for breast (mammography), 12.8% for lung (low dose computed tomography), 13.4% for colorectal (stool-based testing), and 14.5% for cervical (Papanicolaou test) cancer screening [14,15,16,17]. Individuals undergoing screening for multiple cancers using single-cancer tests are exposed to the sum of these false positive rates. Further, the cumulative false positive rate in a multimodal screening program increases for each round of recommended breast, cervical, colorectal, and lung cancer screening, resulting in a significant estimated lifetime risk of unnecessary and potentially invasive diagnostic testing [18]. The risks of false positives resulting from single-cancer screening tests are mitigated somewhat by the well-established and organ-focused diagnostic resolution strategies that follow a positive result. Nevertheless, all false positives result in diagnostic procedures that are unnecessary.

Lung cancer screening programs have targeted only high-risk individuals based on age and smoking history [8], leaving a significant opportunity for early detection and reduced mortality in the general population [11]. However, up to 20% of lung cancers diagnosed annually occur in average-risk non-smokers who, unlike smokers, are more likely to have a histologic diagnosis of adenocarcinoma that progresses more slowly, presenting an opportunity for early detection and successful treatment [19]. 

Lastly, even if additional single-cancer screening approaches were utilized on a population-wide scale, to achieve the necessary performance requirements and demonstrate clinical benefit for lower prevalence cancers, the target population of asymptomatic individuals would be required to perform cancer screening so frequently as to be impractical. Therefore, tests that allow for the early detection of multiple cancers simultaneously are a promising potential solution to address substantial gaps in early cancer detection and screening in order to achieve the Cancer Moonshot goal. 

## 3. Shifting the Screening Paradigm to a Multi-Cancer Approach

A growing interest exists in the development of minimally invasive methods for the early detection of multiple cancers (multi-cancer early detection (MCED) tests). MCED tests may provide an opportunity to improve upon the challenges associated with current single-cancer screening programs. Rather than focusing on single-cancer detection, multi-cancer detection could combine the prevalence of uncommon cancers and evaluate various organ systems using a single diagnostic modality (i.e., blood). By testing for several or many cancers simultaneously in a single blood draw, a multi-cancer approach has the potential to address the gaps in currently available single-cancer screening programs. A minimally invasive blood-based strategy could also address the challenges often encountered with current single-cancer screening approaches, such as test preparation, invasive procedures, lack of access, and low adherence.

Technological innovation over the past decade has facilitated the high-sensitivity analyte detection of various tumor-derived biomarker classes using “liquid biopsies” (blood or other body fluids). Several biomarker classes are considered potential analytes, including whole tumor cells (circulating tumor cells (CTCs)) or the components of tumor cells (cell-free DNA (cfDNA) or cell-free RNA (cfRNA)), as well as targeted proteins and metabolites [20]. To date, single biomarkers as well as combinations of biomarker classes, such as cfDNA mutations, fragmentation, or methylation, and proteins have been extensively investigated for MCED assay development [20]. 

Recent studies of blood-based MCED tests have established their ability to detect a diverse array of cancers across all stages, including cancers with no standard-of-care (SoC) screening approaches [20,21]. These tests show promise in expanding the number of screen-detected cancers if used as an adjunct to established screening paradigms. 

MCED tests may also enable previously unscreened cancers to be diagnosed at earlier stages when there are more effective treatment options, potentially resulting in better outcomes and increased chances of survival [22]. Importantly, some SoC single-cancer screening tests (e.g., the Papanicolaou test for cervical cancer and colonoscopy as well as stool-based tests for colorectal cancer) reduce morbidity and mortality by detecting precancers, facilitating their removal and preventing cancer development. Current blood-based screening tests are not able to detect precancers [23,24].

The success of an MCED screening strategy hinges on its potential to expand access and uptake among the broad range of individuals for whom screening is recommended. Social determinants of health (SDOH) factors, such as race/ethnicity, geographic location, education level, income, insurance coverage status, and access to healthcare, present barriers and influence patient participation in cancer screening adoption and adherence [25,26,27,28]. MCED testing presents a potential opportunity to reduce some of these barriers. Blood draws can be performed in a variety of clinical and community settings, potentially reducing some of the structural barriers associated with cancer screening. However, new technologies, such as MCED testing, may exacerbate disparities by amplifying distrust, fatalism, misperceptions, and inadequate access to further evaluate positive tests in underserved populations. To realize the full potential of MCED testing as a widely adopted and effective screening tool, proactive efforts to ensure equitable access, knowledge, and trust in MCED testing will be needed. If implemented thoughtfully, MCED tests have the potential to expand access to screening in traditionally underserved populations and reduce cancer disparities.

## 4. The Path to Development of a Multi-Biomarker Class MCED Test

Important developmental features need to be considered when developing an MCED test. First is consideration of whether the test should be powered by single- or multi-biomarker classes. While single biomarkers have been extensively utilized and studied for MCED [20,21], a multi-biomarker class approach to MCED testing may enhance the ability to detect the molecular and phenotypic tumor heterogeneity [29,30,31,32]. 

Second, the test should detect multiple cancers, including those representing a substantial burden to public health. For example, lung cancer accounts for approximately 12% of all cancers and 21% of cancer-related deaths in the U.S. [1]. While lung cancer-related deaths are decreasing mostly due to behavioral changes (i.e., smoking cessation) [22], the incidence of lung cancer in non-smokers may be on the rise [33]. Thus, an MCED test with adequate sensitivity for early-stage lung cancer may be a potential solution for those at risk for lung cancer who otherwise would not be eligible for current SoC screening. Likewise, pancreatic cancer accounts for approximately 3% of all cancers in the U.S. and 7% of cancer-related deaths [34]. Pancreatic cancer is a lethal malignancy with a 2% 5-year survival, mainly due to the late presentation of the disease and limited efficacy of available interventions [35]. Pancreatic cancer also lacks SoC screening options. The incidence of pancreatic cancer is expected to rise in Western societies where it is predicted to become the second leading cause of cancer-related death in the U.S. by 2030 [36]. 

**Figure 1:**
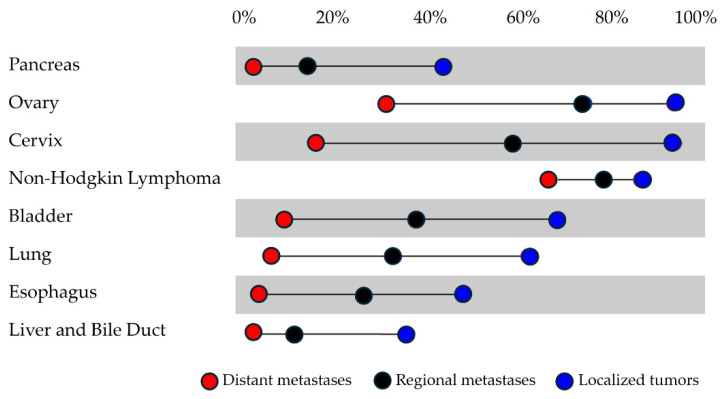
Average 5-year survival rate by stage at diagnosis based on SEER data.

Third, the test should have sufficient sensitivity to detect early-stage cancers when there is a greater chance for curative intervention. Population trends for cancer survival by stage at diagnosis from the Surveillance, Epidemiology, and End Results (SEER) Program reflect that, for many cancers, the chances of survival are greater if cancer is diagnosed early, when it is still localized (Figure 1) [1,37]. 

Fourth, the test should provide high specificity to minimize false positive results. Unlike single-cancer SoC screening tests, blood-based MCED tests interrogate cancer signals originating from multiple organ sites. Thus, a false positive MCED test result may trigger a more complex diagnostic workup to rule out cancer. Therefore, MCED tests are designed with high specificity to minimize false positive results and the associated futile follow-up diagnostic procedures that may impose physical and psychological burdens on patients.

Finally, lower sensitivity is a limitation of MCED tests. While a sensitivity/specificity tradeoff is made when optimizing MCED test performance, most cancer types do not currently have available guideline-recommended screening programs. We anticipate MCED testing synergistically alongside SoC screening will result in more cancers being detected than would have been detected alone. 

The following sections provide an example of one scientific team’s developmental approach for bringing a multi-biomarker class MCED test to broad adoption, from proof-of-concept idea to establishing clinical validation and clinical utility. Table 2 provides the study-related information and test performance characteristics that will be discussed herein. 

### 4.1. Establishing Proof of Concept

A proof-of-concept study for a multi-biomarker class MCED test was performed by Cohen and colleagues, who evaluated an early version of an MCED test (CancerSEEK) [32]. This version of the CancerSEEK test evaluated cfDNA mutations and protein biomarker concentrations, along with machine learning for cancer detection. CancerSEEK utilized a 61-amplicon panel to target 16 genes for cfDNA mutation detection and eight targeted proteins that could discriminate cancer from non-cancer. To ensure high specificity, conventional thresholds were significantly raised for these proteins.

The study evaluated 1005 patients (median age of 64 years) who had previously been diagnosed with eight, organ-specific stage I to III cancers (ovary, liver, stomach, pancreas, esophagus, colorectal, lung, or breast) but did not have metastatic disease nor were being treated prior to testing [32]. 

### 4.2. Establishing Feasibility and Clinical Impact

Leveraging the promise of this new technology, Lennon and colleagues initiated the DETECT-A Study (Detecting cancers Early Through Elective mutation-based blood Collection and Testing), the first prospective, interventional feasibility study in evaluating an MCED test. The DETECT-A study used an MCED test, CancerSEEK, which evaluated cfDNA mutations and proteins without machine learning to screen 10,006 women (65–75 years of age) with no history of cancer [31]. This version of CancerSEEK utilized a two-stage testing procedure, a baseline test followed by a confirmatory test in participants whose baseline test was positive. The confirmation test was designed to ensure that detected mutations were not due to clonal hematopoiesis of indeterminate potential (CHIP). Simultaneous intravenous contrast positron emission tomography–computed tomography (IV PET-CT) imaging was incorporated to evaluate positive MCED test results and rule out or localize cancers. 

The authors reported that the CancerSEEK testing strategy detected 26 individual cancers with a sensitivity of 27.1% and a specificity of 98.9%. Sensitivity was calculated as an empiric estimate of sensitivity, with all cancers diagnosed by any means in a 12-month period as the denominator. CancerSEEK identified cancers at all four stages, and 65% of Cancer-SEEK detected cancers were confined to localized or regional disease [31]. 

Currently, the DETECT-A is the only prospective study of an MCED test that has reported longitudinal outcomes for individuals screened with the test over a median of 4.3 years [41]. Preliminary analyses reported for this longitudinal cohort confirmed that all patients diagnosed with and treated for stage I and II cancers achieved and had maintained remission. Importantly, more than one-half of patients diagnosed with stage III cancer by the MCED test remained alive, and 60% of those alive were also cancer-free. Of the patients identified with cancer through MCED testing, 54% were surgically treated and, of those treated, 86% remain alive and cancer-free. Though the event rate is low and the follow-up time short, these early results demonstrate that MCED testing has potential utility in detecting early cancers amenable to treatment with curative intent. 

When developing an MCED test, maintaining a high specificity is important to minimizing patient harms. In the DETECT-A study by Lennon et al., the rate of false positives, defined as individuals with a positive MCED test result but who were found to have no evidence of malignancy following an imaging-based diagnostic workup and 12 months of follow-up, was approximately 1% [31]. The preliminary findings of a prospectively planned analysis of cancer incidence among DETECT-A participants with false positive results found that 96.4% of false positive patients remained cancer-free for more than four years following their enrollment in the study [42]. The incidence of cancer was relatively low and consistent with the annual incidence rate for average risk women aged 65–74 years [43]. 

### 4.3. Biomarker Selection and Classifier Development 

Following DETECT-A, several subsequent case–control studies have evaluated the ability to expand the multi-marker approach to other biomarker classes in order to improve sensitivity while maintaining specificity [38,40,44]. Aneuploidy, or chromosome copy number variations, was the first genomic abnormality identified in cancer [45,46] and is prevalent in the majority of cancers [47]. Douville and colleagues evaluated aneuploidy, in addition to mutations and proteins, in 883 patients with eight established non-metastatic cancers (ovarian, colorectal, esophageal, liver, lung, pancreatic, stomach, and breast) [30]. Aneuploidy was detected in 49% of these non-metastatic cancer samples, and the combination of aneuploidy with mutations and proteins resulted in increased overall sensitivity. 

Assay of methylation patterns in cfDNA is a powerful complement or alternative to mutations and proteins for liquid biopsy application in MCED. Aberrant methylation patterns in the DNA from neoplastic cells differ from those found in healthy cells and have been developed into highly informative biomarkers for numerous cancer types [48]. Additionally, tumor-related (hyper)methylation tends to occur in specific regions of the genome, which are highly consistent across individuals and clinical stages (including pre-cancers), thus making it amenable to less costly polymerase chain reaction (PCR)-based platforms utilizing smaller marker panels. Because methylation is an early event in oncogenesis, it has been shown to be broadly informative for the detection of pre-cancers and cancers in a variety of single organ systems; a clear example is the multi-target stool DNA test (Cologuard^®^, Exact Sciences, Madison, WI, USA), which is FDA-approved for average risk colorectal cancer screening in 2014 [14]. This test assays methylated *BMP3* and *NDRG4*, in addition to mutant *KRAS* and hemoglobin, and has been used for colorectal cancer screening in over 15 million individuals. Several other groups have investigated both targeted PCR for methylation markers (Guardant Health, Palo Alto, CA, USA) and next-generation sequencing assays for methylation (GRAIL, Menlo Park, CA, USA) in MCED applications [23,49]. 

Katerov and colleagues evaluated methylated cfDNA markers (MDMs) along with select proteins in previously published in single-cancer clinical pilot studies [44,50]. A 2023 study included 146 controls and 236 cases encompassing thirteen cancer sites (bladder, breast, cervical, colorectal, esophageal, kidney, liver, lung, ovarian, pancreatic, prostate, stomach, and uterine). At 99% specificity, the overall sensitivity for cancer detection was 68% for MDMs, 43% for proteins, and 75% for MDMs and proteins combined [44]. In a recent multi-class biomarker feasibility study, Gianullin and colleagues evaluated a three- and four-biomarker class approach while leveraging the analytical improvements and learnings from the aforementioned studies [51]. The study included 3518 samples divided into a training and validation set (cancers: 1821; non-cancers: 565) and a test set (cancers: 566; non-cancers: 566). The training and validation set evaluated three biomarker classes (proteins, methylation, and aneuploidy), while the test set evaluated and compared the three-biomarker class to a four-biomarker class (proteins, methylation, aneuploidy, and mutations). Specifically, the test set included 15 cancer sites (lung, breast, colon, uterine, kidney, pancreatic, prostate, bladder, stomach, esophageal, liver, ovarian, uterine, non-Hodgkin lymphoma, and multiple myeloma). The three- and four-biomarker classes exhibited overall sensitivities of 53.4% and 61.0% and specificities of 98.8% and 98.2%, respectively. At >98% specificity, the sensitivity of the four-biomarker class test was 38.7% for stage I and II cancers, 10.1% higher than that of the three-biomarker class test. This evidence demonstrated that multi-biomarker class MCED configurations have the potential for robust sensitivity in detecting a broad range of cancer types and stages at high specificity.

While preliminary evidence suggests that a multi-class biomarker approach to an MCED test enhances sensitivity, combining multiple biomarker assays within a single test may also increase assay complexity and costs and reduce specificity. 

### 4.4. Assessing Performance in Analytical Validation

Most recently, two biomarker classes, methylation and protein, have been rigorously evaluated in samples prospectively collected from *Ascertaining Serial Cancer patients to Enable New Diagnostic 2* (ASCEND 2), a large, multi-center study [39,40]. This two-biomarker performance study assessed 6314 samples divided into a training set (cancers: 654; non-cancers: 2372) and a test set (cancers: 772; non-cancers: 2516). Cancer stages were well-represented across 21 solid and hematologic tumor organ sites representing >85% of incident cancers. At 98.5% specificity, the two-biomarker class assay demonstrated an overall sensitivity of 54.8% in cancer organ types without average-risk SoC screening (i.e., excluding breast, prostate, cervical, and colorectal) and 63.7% sensitivity for the six most aggressive cancer organ types with the shortest 5-year survival rate (i.e., pancreatic, esophageal, liver, lung and bronchial, stomach, and ovarian) [40]. Overall, this study demonstrated robust performance of a multi-biomarker class MCED test utilizing methylation and protein biomarkers. 

### 4.5. Establishing Clinical Effectiveness and Implementation in Clinical Validation and Clinical Utility

Rigorous evidence is vital to the advancement and eventual adoption of MCED tests. The successes of the aforementioned studies along the developmental pathway have paved the way for a rigorous pivotal trial and real-world evidence studies to evaluate the impact of a multi-biomarker class test in clinical practice (Table 3). To this end, the pivotal study, *Detecting Cancers Earlier Through Elective Blood-Based Multi-Cancer Early DeTection (MCED) testing—Study of All ComeRs* (DETECT-SOAR), is planned as a prospective, randomized, controlled, open-label, interventional trial to evaluate the safety and effectiveness of the multi-biomarker class test. 

Lastly, real-world evidence (RWE) collection is planned through the Falcon Registry Study to evaluate the impact of the test in routine clinical practice and to establish a data repository for future development efforts and collaborations. This RWE will enable the creation of a research database platform aimed towards establishing RWE. RWE studies will facilitate rigorous investigation into the clinical, operational, and economic impact of the testing in routine clinical practice.

### 4.6. Clinical Implementation of MCED Testing and Diagnostic Resolution 

More research is warranted in clinical validation studies to support the feasibility and safety of integrating MCED tests in clinical practice [31,49,52]. One issue in the clinical implementation of MCED testing is the ideal pathway to diagnostic resolution after a positive MCED test. This area remains largely unexplored, highlighting the need for further investigation into its implications and potential optimizations. 

**Figure 2:**
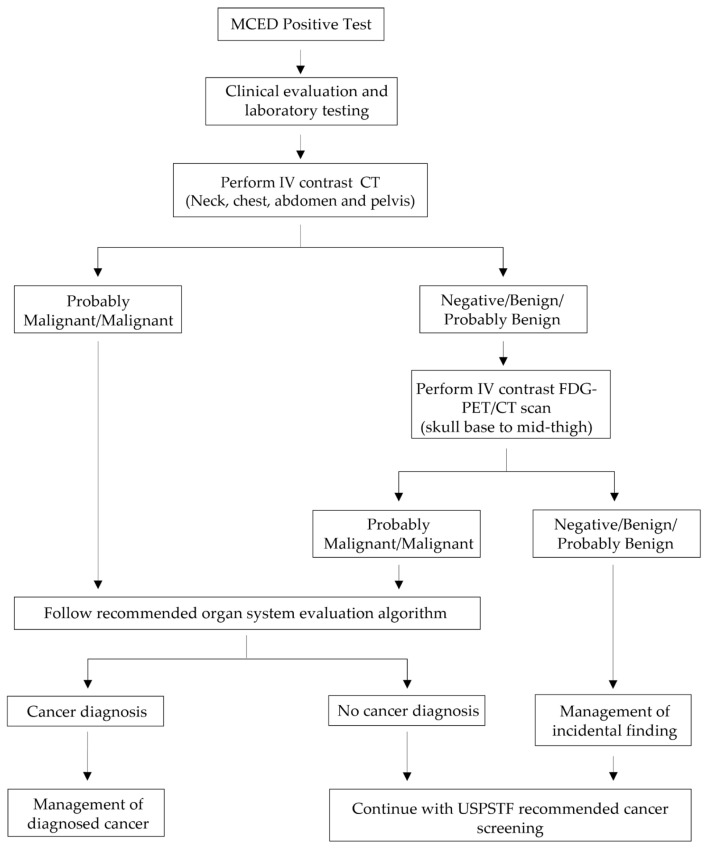
Proposed clinical algorithm for diagnostic resolution through imaging following an MCED test. * Abbreviations: CT: Computed Tomography; FDG: Flourine-18 Fluorodeoxyglucose; MCED: Multi-Cancer Early Detection; PET: Positron Emission Tomography; USPSTF: United States Preventive Services Task Force. * This clinical algorithm is not meant to replace clinical standard of care. Suggested pathways are intended to encourage clinicians to ask questions and consider important factors as they define the ideal diagnostic strategy for their patients.

Here, a clinical algorithm for diagnostic resolution through imaging following an MCED test (Figure 2) is proposed for tests not returning information on the tissue of origin from which the cancer signal originates. If a participant receives a negative MCED test result, they should continue with USPSTF-recommended cancer screening. However, if the participant receives a positive MCED test result, a clinical evaluation with screening questions should be performed, along with laboratory testing including a complete blood count and a basic metabolic panel, with clinically indicated diagnostic evaluation (Table S1). If this evaluation is negative for cancer, computed tomography (CT) imaging with intravenous (IV) contrast (neck, chest, abdomen, and pelvis) should be completed. If imaging shows probable malignancy/malignancy, then the provider should follow a recommended organ system evaluation algorithm to achieve a final diagnosis (Table S2). If the imaging is negative or reveals a benign/probably benign status, the provider should consider common non-cancer findings (Table S3) and the participant should have flourine-18 fluorodeoxyglucose positron emission tomography/computed tomography (FDG-PET/CT) imaging (skull base to mid-thigh) performed. If the results of the imaging are probably malignant/malignant, then the provider should follow a recommended organ system evaluation algorithm to achieve a formal diagnosis. If negative/benign/probably benign, the provider should consider management of incidental findings (Table S4). If the participant is ultimately determined to be cancer-free, then repeat MCED testing can be considered at 12 months. An imaging-based strategy such as this has been shown to be 28% more efficient than a molecular-based strategy for tumor localization in modeling studies evaluating diagnostic efficiency of different approaches [53]. This proposed diagnostic approach needs to be validated in prospective studies. Other test manufacturers are developing distinct diagnostic strategies and some incorporate molecular predictors of the location of suspected cancers. These approaches are also awaiting validation in prospective trials.

## 5. Core Concepts for Consideration during MCED Test and Study Design

Several core concepts need to be considered for MCED test and study design. With respect to test design, it is imperative to balance both the benefits and harms of the MCED test [54]. The benefits include detection of a number of clinically significant cancer types [31], detection of cancers at early stages when they may be more amenable to curative intent, and ultimately reducing cancer-related mortality [55,56]. Conversely, there are a number of potential harms of MCED testing that are important to consider, including the false positive rate, overdiagnosis, and overtreatment. 

MCED tests are being designed to limit false positive rates to avoid diagnostic odysseys involving unnecessary imaging procedures and radiation exposure that may result in patient harm and test-related anxiety. The false negative rate could also be viewed as a potential harm; however, patients would be reassured of their MCED test result if they continue recommended SoC screening and receive annual physical exams to monitor their health. Future prospective, longitudinal studies involving annual testing will explore the impact of false negatives more deeply. 

Detection of clinically insignificant cancer or pre-cancer, also known as overdiagnosis, may lead to overtreatment and is a variably important challenge in cancer screening. Overdiagnosis is less of a concern for colorectal and cervical screening, as pre-cancers can be removed with low risk of clinical complications. However, for prostate, lung, and breast cancer screening, overdiagnosis has been a source of concern. To date, available data from prospective studies of MCED suggest that pre-cancer is very rarely detected and sensitivity for stage I cancer is relatively low, making the detection of small, clinically insignificant cancers relatively unlikely [31,49]. Future studies will provide additional results on this topic, but DNA based biomarkers appear to be released into the bloodstream preferentially by more aggressive or larger cancers, making overdiagnosis less likely with this screening modality.

From a study design perspective, one of the most important considerations is the differential test performance characteristics related to diagnostic accuracy across study types. Case–control studies are designed to include a variety of predefined cases; in this context, selected cancer types and stages of interest, as well as a predefined cohort of controls (non-cancer cases). Most case–control studies include cases and controls of similar age, gender, ethnicity, race, and overall health. Therefore, notable advantages exist when utilizing case–control studies compared to prospective, cohort studies in the initial MCED test development and validation setting [54]. Since cancer status is known, case–control studies can avoid the time and costs associated with the long-term follow up of all study participants. In addition, the ratio of cases to controls is enriched in case–control studies, so they are particularly efficient in estimating assay performance for less prevalent and rare cancers where prospective cohort studies would require large numbers of participants to accrue adequate numbers of incident cases to be sufficiently powered [55]. While case–control study data are valuable, test performance varies depending on the selection of both the cases and controls [56] and specific test attributes. 

Conversely, spectrum bias, or the disproportionate sampling of cases and/or controls to include predominately the extreme ends of the disease spectrum (cancer and healthy), is common in case–control studies and can inflate test accuracy estimates [54]. In general, test sensitivity increases with stage, so case–control studies that include a disproportionate number of advanced stage (III/IV) cancer cases will likely result in higher overall sensitivities than those that focus on the inclusion of predominately early-stage cancer cases. MCED test performance also varies widely across individual cancer types [20], so studies that disproportionately include specific cancer types with high test sensitivities (liver, ovarian, colorectal) while excluding those with low sensitivities (breast, prostate, thyroid) will exhibit higher overall sensitivities. Additionally, technical issues such as sample collection, storage/processing conditions, and specific assay attributes influence MCED test results [57]. Study design and MCED-test-specific attributes likely contribute to the wide range of case–control sensitivities reported to date [21]. 

Importantly, test performance in case–control studies is not representative of what would be expected in an intended-use screening population. Clinical validation studies must be confirmed at the population level, with prospective studies including participants reflecting the intended-use population to demonstrate clinical utility. Therefore, the MCED test sensitivity in prospective studies has been lower than that observed for case–control studies (i.e., a reported sensitivity of 51.5% in GRAIL’s CCGA (case–control) study compared to the 28.9% (early test version) or 20.8% (marketed test version) sensitivity in PATHFINDER) [49,58]. 

## 6. Conclusions

Innovative technologies incorporating single- and multi-biomarker classes into blood-based MCED tests are integral to achieving the Cancer Moonshot initiative of reducing mortality from cancer. Early evidence suggests that such testing can detect clinically significant cancers that impact public health and also detect them at early stages when they are more amenable to curative interventions. Rigorous trials and real-world evidence studies are warranted to evaluate the safety and effectiveness of testing in various populations and the clinical, operational, and economic impact of the testing in routine clinical practice.

## Figures and Tables

**Table 1:** USPSTF-endorsed screening programs.

	Breast [9]	Colorectal [7,10]		Lung [8]	Cervical [6]
Population	Average risk *	Average risk		High risk	Average risk
Recommendation Level **	B	B	A	B	A
Age at First Screening, y	40	45–49	50–75	50 ***	21
Screening Modality	Mammography Ultrasound MRI	Stool-based methods FIT HS-gFOBT mt-sDNA Direct visualization Colonoscopy Flexible sigmoidoscopy CT colonography		Low-dose CT	Pap test HPV test
Interval for testing, y	1–2	1–10 ^†^		1	3–5

Abbreviations: USPSTF: US Preventive Services Task Force; MRI: magnetic resonance imaging; FIT: fecal immunochemical test; mt-sDNA: multi-target stool DNA; HS-gFOBT: high-sensitivity guaiac-based fecal occult blood test; CT: computed tomography; HPV: human papillomavirus; Pap: Papanicolaou; y: years. * Women. ** USPSTF recommendations: A = The USPSTF recommends the service. There is high certainty that the net benefit is substantial; B = The USPSTF recommends the service. There is high certainty that the net benefit is moderate or there is moderate certainty that the net benefit is moderate to substantial. *** If patient meets high risk criteria (at least 20 pack-year smoking history). ^†^ Depending on test result.

**Table 2:** Developmental evidence to support a multi-biomarker class MCED test.

	Lennon et al. [31]	Douville et al. [38]	Douville, et al. [39] Gainullin et al. [40]
Study Name	DETECT-A	Biomarker Feasibility	ASCEND-2
Study Type	Prospective interventional	Case–control	Case–control
N	10,006	3518 Training and validation set (Cancers: 1821; Non-Cancers: 565) Test set (Cancers: 566; Non-Cancers: 566)	6314 (Cancer: 1426; Non-Cancer: 4888)
Population	Asymptomatic women aged 65–74 years with no history of cancer	Men and women	Men and women aged >/ = 50 years
Biomarker Classes	Mutations Proteins	Mutations Proteins Aneuploidy Methylation	Proteins Methylation
No of Cancers Detected (Organ types)	26 (ovarian, lung, uterine, thyroid, colorectal, breast, lymphoma, kidney, appendix, carcinoma of unknown primary)	15 (breast, bladder, colorectal, esophageal, kidney, liver and bile duct, lung and bronchus, ovarian, pancreatic, prostate, stomach, uterine, non-Hodgkin’s lymphoma, multiple myeloma, myelodysplastic syndrome)	21 (anal, bladder and urinary, breast, cervical and uterine, colorectal, esophageal, head and neck, kidney, liver and bile duct, lung and bronchial, multiple myeloma, non-Hodgkin lymphoma, ovary, pancreatic, prostate, small intestinal, stomach, testicular, thyroid, uterus, vulva)
Sensitivity, %	27.1%	3 marker classes: 55.2% 4 marker classes: 62.4%	50.9%
Specificity, %	98.9%	3 marker classes: 99.0% 4 marker classes: 98.0%	98.5%
False Positive Rate, %	1.0%	n/a	n/a

Abbreviations: DETECT-A: Detecting cancers Early Through Elective mutation-based blood Collection and Testing; ASCEND: Ascertaining Serial Cancer patients to Enable New Diagnostic 2.

**Table 3:** Current and future studies of a multi-biomarker MCED test.

Evidence Aim	Studies	Goal
Clinical Feasibility	DETECT-A—A prospective interventional trial to establish MCED characteristics.	To evaluate the impact of an MCED test on cancer downstaging, patient compliance with SoC screening, and patient perceptions.
Analytical Validation	ASCEND-2—A multi-center, prospectively collected case–control study.	To assess the performance of a refined MCED test classifier algorithm using samples from adults ≥50 years old of all genders with known cancer, suspicion of cancer, and controls without cancer.
Clinical Validity and Clinical Utility	DETECT-SOAR—A prospective, randomized, controlled, open-label, intervention pivotal trial.	To evaluate the safety and effectiveness of MCED testing in the indicated population (adults of all genders aged 50–84 years).
Clinical Utility	FALCON—A RWE registry evaluating clinical outcomes, treatment patterns, healthcare utilization patterns, and economic burden among patients who have undergone MCED testing.	To evaluate if MCED testing can inform clinical management.

Abbreviations DETECT-A: Detecting cancers Early Through Elective mutation-based blood Collection and Testing; ASCEND: Ascertaining Serial Cancer patients to Enable New Diagnostic 2; SOAR: Detecting Cancers Earlier Through Elective Blood-Based Multi-Cancer Early DeTection (MCED) testing—Study of All ComeRs (SOAR); RWE: Real-World Evidence; SoC: Standard of Care; MCED: Multi-Cancer Early Detection.

## Data Availability

Not applicable.

## Supplementary Materials

The following supporting information can be downloaded at: https://www.mdpi.com/article/10.3390/life14080925/s1, Table S1. Examples of (a) Participant History and (b) Laboratory Testing Following a Positive MCED Test; Table S2. Recommended Organ System Evaluation Algorithm Following CT with IV Contrast or FDG-PET/CT Result of Probably Malignant/Malignant [59,60,61,62,63,64,65,66,67,68,69,70,71,72,73,74,75,76,77]; Table S3. Common Non-Cancer Findings by Organ System Following FDG-PET/CT with IV Contrast Result of Negative/Benign/Probably Benign; Table S4. Common Incidental Finding Evaluation by Organ System Following CT or FDG-PET/CT.
