# Supplementary material for: Shifting the Cancer Screening Paradigm: Developing a Multi-Biomarker Class Approach to Multi-Cancer Early Detection Testing
Source: Life (Basel). 2024 Jul 24;14(8):925. doi: 10.3390/life14080925 (PMC11355654; doi:10.3390/life14080925)
Supplement: Supplementary file 1 [file life-14-00925-s001.zip › life-3033196-supplementary.pdf]

Shifting the Cancer Screening Paradigm: Developing a Multi-biomarker Class Approach to Multi-Cancer Early Detection Testing

Supplemental Tables

Table S1. Examples of (a) Participant History and (b) Laboratory Testing Following a Positive MCED Test

a.

**System**	**Examples of Symptoms of Concern**
General	Persistent constitutional symptoms such as fatigue, malaise, fevers, night sweats, unexplained weight loss.
Abdominal	Persistent unexplained abdominal pain nausea, bloating, loss of appetite.
Bowel	Persistent changes in bowel habits (diarrhea, constipation, blood in stool).
Respiratory	Persistent unexplained respiratory symptoms such as cough, shortness of breath, hemoptysis, pain with respiration.

b.

	**Cancer Type**	**Laboratory Test**	**Laboratory Results**	**Interpretation**
Hematological	Leukemia	CBC	RBCs, WBCs, and platelets	Elevated WBCs typically suggest further evaluation for leukemia. Abnormalities in RBCs and platelets are non-specific by may suggest leukemia or other hematologic neoplasms
	Multiple Myeloma	BMP and CBC	Calcium levels	Hypercalcemia, elevated creatinine, and anemia can be suggestive of multiple myeloma

	Myeloproliferative Disorders	CBC	RBCs, WBCs, platelets	Abnormal elevations in WBCs, RBCs, and or platelets can be suggestive of myeloproliferative disorders

Abbreviations: CBC: Complete Blood Count; RBCs: red blood cells; WBC: white blood cells; BMP: Basic Metabolic Panel

Table S2. Recommended Organ System Evaluation Algorithm Following CT with IV Contrast or FDG-PET/CT Result of Probably Malignant/Malignant

Organ System	Diagnostic Approach
Respiratory [59-61]	• Lung (peripheral): CT-guided biopsy<br>• Lung (central): Pulmonary consultation for bronchoscopic-guided biopsy
Digestive [62,63]	• Esophago-gastric: Upper endoscopy<br>• Small intestine: Gastroenterology consult<br>• Colon: Colonoscopy<br>• Appendix: Surgery consultation<br>• Gallbladder: Gastroenterology consultation<br>• Pancreas: Gastroenterology consultation
Circulatory [64-66]	• Cardiac/Vascular surgery consultation
Urinary [64,67,68]	• Kidney/Upper Tract: Ultrasound-guided biopsy vs. urology consultation<br>• Lower Tract: CT urogram/cystoscopy
Integumentary (skin, fat) [69]	• Subcutaneous: US-guided Biopsy<br>• Cutaneous: Biopsy or dermatology consultation
Skeletal [64]	• Ultrasound-guided biopsy
Muscular [70]	• Ultrasound-guided biopsy
Endocrine [64,71,72]	• Thyroid/Parathyroid: Neck ultrasound +/- endocrinology consultation<br>• Adrenal: Biochemical evaluation +/- endocrinology consultation
Lymphatic [64,66,73]	• Lymph node: Ultrasound-guided biopsy<br>• Spleen: Hematologic laboratory evaluation or surgery consultation for solitary lesions
Nervous System [74]	• Neurology/neurosurgery consultation
Reproductive System [75-77]	• Breast: Ultrasound-guided biopsy<br>• Ovary/Uterus: Gynecology consultation<br>• Prostate: PSA and prostate US/MRI<br>• Testicle: Urology consultation

Abbreviations: CT: Computed Tomography; IV: Intravenous; PSA: Prostate Specific Antigen; US: Ultrasound; MRI: Magnetic Resonance Imaging: PET: Positron Emission Tomography

This guidance is not meant to replace clinical standard of care. Suggested pathways are intended to encourage clinicians to ask questions and consider important factors as they define the ideal diagnostic strategies for their patients.

Table S3. Common Non-Cancer Findings by Organ System Following FDG-PET/CT with IV Contrast Result of Negative/Benign/Probably Benign

Organ System	Common Non-Cancer Findings
Respiratory	• Vocal cord nodules/paralysis<br>• Infection/inflammation<br>• Pulmonary nodules<br>• FDG micro-emboli
Digestive	• Diverticulitis<br>• Benign ileocecal uptake<br>• Infection<br>• Liver cyst, hemangiomas, cirrhosis<br>• Gallbladder calculi, cholecystitis
Circulatory	• Aneurysm<br>• Atherosclerosis
Urinary	• Physiologic urine uptake<br>• Adrenal nodule<br>• Cyst<br>• Calculi<br>• Focal prostatitis
Integumentary (skin, fat)	• Seborrheic dermatitis<br>• Actinic keratosis
Skeletal	• Hemangiomas<br>• Bone island
Muscular	• Lipoma<br>• Bursitis<br>• Calcifications
Endocrine	• Thyroid cyst, nodules<br>• Adrenal nodules<br>• Adenopathy
Lymphatic	• Lymphadenopathy
Nervous System	• Pituitary incidentalomas<br>• Rathke's cleft cyst

Reproductive System	• Adnexal: Premenopausal status - Mid-ovulatory cycle<br>• Uterine: Premenopausal status - Fibroids (leiomyoma)<br>• Prostate: Recent biopsy - Prostatitis<br>• Breast: Postpartum/lactation – Mastitis, gynecomastia

Abbreviations: CT: Computed Tomography; IV: Intravenous; FDG: Flourine-18 Fluorodeoxyglucose; PET: Positron Emission Tomography

This guidance is not meant to replace clinical standard of care. Suggested pathways are intended to encourage clinicians to ask questions and consider important factors as they define the ideal diagnostic strategies for their patients.

Table S4. Common Incidental Finding Evaluation by Organ System Following CT or FDG-PET/CT

Organ System	Common Incidental Finding Evaluation
Respiratory	• Fleischner Criteria Guidance
Digestive	• Upper aerodigestive tract<br>o Fiberoptic endoscopy +/- biopsy<br>o Clinical evaluation at 3 months & radiological follow-up at 6 months<br>• Lower gastrointestinal tract<br>o Colonoscopy +/- biopsy
Circulatory	• Ultrasound for aneurysm<br>• Evaluation for atherosclerosis as indicated<br>• Vascular surgery consultation as indicated<br>• Cardiology consultation as indicated
Urinary	• Ultrasound or MRI<br>• Urology consultation as indicated
Integumentary (skin, fat)	• Skin biopsy<br>• 6-month evaluation<br>• Dermatology consultation as indicated
Skeletal	• MRI with and without intravenous gadolinium for hemangioma confirmation<br>• Imaging and 6-month follow up for bone island
Muscular	• Ultrasonography<br>• Biopsy as indicated
Endocrine	• Thyroid<br>o Incidental thyroid nodules follow-up with ultrasonography<br>o Suspicious or larger nodules: ultrasound-guided FNAC<br>o Thyroid function testing<br>• Parathyroid<br>o Sestamibi scan<br>o Endocrinology consultation as indicated<br>• Adrenal<br>o Hormonal evaluation<br>o Endocrinology consultation as indicated
Lymphatic	• Hematologic lab evaluation and ultrasound<br>• FNAC, core needle biopsy or excisional biopsy for histopathological evaluation
Nervous System	• Hormonal evaluation and MRI at 6 months (macro incidentalomas) and 1 year (micro incidentalomas)<br>• Visual field evaluation

<table>
<tr><td></td><td>Neurology consultation as indicated
FNAC, core needle biopsy or excisional biopsy for histopathological evaluation</td></tr>
<tr><td>Reproductive System</td><td>Breast
Ultrasound-guided core needle biopsy
Cystic lesions resolving after FNAC requires no further evaluation
If clinical breast examination, imaging and final needle aspiration reveals benign disease, repeat clinical breast examination at 4 to 6 weeks
Uterine
Clinical and radiological evaluation as indicated
Gynecology consultation as indicated
Adnexal
Ultrasonography
Pre-menopausal
Simple cyst < 5 cm = no further action
Simple cyst > 5 cm = Repeat ultrasonography at 6 to 12 months
Post-menopausal
Ultrasound at 6 to 12 months
MRI for complex masses or indeterminate on ultrasound
Prostate
Ultrasound
PSA</td></tr>
</table>

Abbreviations: CT: Computed Tomography; FDG: Flourine-18 Fluorodeoxyglucose; PET: Positron Emission Tomography; FNAC: Fine Needle Aspiration Cytology; MRI: Magnetic Resonance Imaging; PSA: Prostate-Specific Antigen

This guidance is not meant to replace clinical standard of care. Suggested pathways are intended to encourage clinicians to ask questions and consider important factors as they define the ideal diagnostic strategies for their patients.
